# Duration of ADHD medication treatment among Finnish children and adolescents ‒ a nationwide register study

**DOI:** 10.1007/s00787-025-02735-4

**Published:** 2025-05-07

**Authors:** Terhi A. Kolari, Miika Vuori, Hanna Rättö, Eveliina A. Varimo, Eeva T. Aronen, Kari Auranen, Leena K. Saastamoinen, Päivi T. Ruokoniemi

**Affiliations:** 1 Department of Biostatistics and Turku University Hospital, University of Turku, Turku, Finland; 2 The Social Insurance Institution of Finland, Research Unit, Helsinki, Finland; 3 Department of Public Health, University of Turku, Turku, Finland; 4 INVEST Research Centre, University of Turku, Turku, Finland; 5 Pediatric Research Center, New Children’s Hospital, Helsinki University Hospital, Helsinki, Finland; 6 University of Helsinki and Helsinki University Hospital, Child Psychiatry, Helsinki, Finland; 7 Department of Mathematics and Statistics, University of Turku, Turku, Finland; 8 Department of Clinical Medicine, University of Turku, Turku, Finland; 9 Information and Development Services, Finnish Medicines Agency, Helsinki, Finland; 10 Department of Clinical Pharmacology, University of Helsinki, Helsinki, Finland; 11 The Individualized Drug Therapy Research Program (INDIVIDRUG), University of Helsinki, Helsinki, Finland; 12 Department of Biostatistics, University of Turku, Turku, 20014 Finland

**Keywords:** ADHD, Medicine use, Treatment duration, Population based, Registry study, Reimbursement data

## Abstract

**Aim:**

To study the duration of attention-deficit/hyperactivity disorder (ADHD) medication treatment among children and adolescents by sex and age group in Finland during 2008–2019.

**Methods:**

This was a descriptive, population-based register study covering all Finnish children and adolescents aged 6–18 years who initiated their first ADHD medication treatment period between January 1, 2008 and December 31, 2019 (*n* = 40691). To establish the duration of use we collected data from the register of Dispensations reimbursable under the National Health Insurance Scheme register. The median follow-up time was 3.8 years (Q1 = 1.7, Q3 = 7.1). Treatment duration was calculated as the interval between the date of the first and last purchase with a cut-off of 365 days allowed between purchases. The durations were estimated using Kaplan-Meier survival times.

**Results:**

The median duration of ADHD medication treatment was 3.2 years (95% CI 3.2, 3.3, Q1 = 1.0 95% CI 0.9, 1.0, Q3 = 6.8 95% CI 6.7, 7.0). Sex and age significantly influenced treatment duration (*p* <.0001 and *p* <.0001). Boys had longer treatment duration than girls and the younger the subject, the longer the duration of usage. Boys aged 6–8 years (32.4% of the subjects) exhibited the longest treatment duration with a median of 6.3 years (95% CI 6.2, 6.5, Q1 = 2.6 95% CI 2.5, 2.7, Q3 = 9.4 years 95% CI 9.2, 9.6).

**Conclusions:**

The duration of ADHD medication treatment among children in the real-world clinical setting goes well beyond the data available from randomized controlled trials and extends for several years especially among young boys.

**Supplementary Information:**

The online version contains supplementary material available at 10.1007/s00787-025-02735-4.

## Introduction

Attention deficit hyperactivity disorder (ADHD) is a common neurodevelopmental disorder, including persistent symptoms of inattention, hyperactivity, and impulsivity that interfere with cognitive and social functioning [1]. According to community and administrative prevalence studies, ADHD affects around 3–7% of the pediatric population [2–4]. The majority of children and adolescents have onset between 7 and 16 years of age [5]. Longitudinal studies suggest that persistence rates of pediatric ADHD into adulthood are around 50% [6]. However, follow-up studies suggest that persistence rates could be higher, in particular, among clinic-referred children with co-occurring psychiatric disorders [7]. These children are also likely to display fluctuating levels of persistence and remission from childhood to adulthood.

Randomized, placebo-controlled efficacy studies have rigorously demonstrated the efficacy of ADHD medications in short-term treatment (i.e. 12 weeks) of ADHD core symptoms in children and adolescents [8, 9]. Naturalistic large database studies indicate that medical treatment for pediatric ADHD may decrease the risk of harmful outcomes, such as injuries, poor academic performance and reactive aggression, compared with untreated ADHD [10–13]. However, some studies with causal inference approaches have found less evidence on ADHD medication effects on psychological well-being and injuries [14, 15]. Register-based studies are also prone to confounding [10, 16]. Therefore, the long-term benefits of ADHD treatment remain uncertain [17], especially when studies employing enrichment designs (e.g. trials focusing solely on drug responders) are not accounted for [9, 18]. Also, the research on long-term safety issues with ADHD medication use is scarce [19].

The Finnish Current Clinical guideline on the treatment of ADHD recommends medication to be considered for all children with a diagnosis of ADHD and aged 6 years and over [20]. The preferred first choice for medicating pediatric ADHD is methylphenidate [20]. According to a recent Cochrane review [21], methylphenidate does not seem to cause serious life-threatening adverse events in childhood or adolescence when used for up to six months. Methylphenidate may, however, be associated with an increased risk of non-serious adverse events, such as sleep problems and decreased appetite. Nonetheless, the Cochrane study group evaluated the quality of evidence for all outcomes to be very low and the true magnitude of both positive and negative long-term effects to be unclear. The lack of randomized, placebo-controlled trials evaluating potential long-term effects of ADHD medications has been acknowledged also previously [9, 22].

As the prevalence of ADHD medication use has increased notably among children and adolescents during the last decades [23], the safety and effectiveness of treating pediatric ADHD with medicines has become an important public health concern in many countries [19, 24]. Increased ADHD medicine use in real-life clinical settings may also relate to harmful effects in the pediatric population such as deterioration in academic performance [14] and there is current debate on whether long-term use of stimulant medication relates to decreased height velocity [19, 22, 25, 26]. On the other hand, long-term adherence to ADHD medications is known to decrease, reducing the risk of harmful, medication-related adverse effects in the long run. In a follow-up of the Multimodal Treatment Study of Children with ADHD, only 32.5% of the subjects initially enrolled, were 50% adherent to medication at 8 years compared to 63.3% at 14 months [27].

There has been a lack of population-based studies that examine ADHD medication treatment duration in children and adolescents in more detail. Some earlier studies have indicated that approximately 50 to 75 per cent of children and adolescents remain on treatment after one year [28–31]. However, a very recent, multinational observational study found that across the nine countries studied, treatment persistence among children (4–11 years) was 50–60% for up to five years of follow-up [32]. In addition, the observed discontinuation rates were higher among adolescents (12–17 years) as compared to children and patterns were similar across sex [32]. Nonetheless, we are not aware of up-to-date data on treatment duration going beyond the 5 years of follow up and with a more in-depth analysis of age at initiation affecting persistence.

In this study, we aimed to investigate ADHD medication treatment duration among Finnish children and adolescents initiating ADHD medication between the years 2008 to 2019. To address the knowledge gaps, we analyzed the duration of the first ADHD medication treatment period for children within age groups of 6–8 and 10–12 years at initiation, and for adolescents within 13–15 and 16–18 years, respectively.

## Methods

### Data

This was a descriptive study based on the nationwide administrative registers maintained by the Social Insurance Institution of Finland (SII) [33–35]. All permanent residents in Finland are included in the National Health Insurance (NHI) system. We collected data on all ADHD drug purchases from the register of dispensations reimbursable under the NHI scheme, which includes records of all reimbursed purchases from Finnish pharmacies. ADHD drug purchases were identified by referring WHO Anatomical Therapeutic Chemical (ATC) classification codes N06BA02 (dexamfetamine), N06BA04 (methylphenidate), N06BA09 (atomoxetine), N06BA12 (lisdexamfetamine) and C02AC02 (guanfacine) [36]. These are the only medications with regulatory approval in the treatment of ADHD in Finland. For each purchase, the dispensing date, pseudonymized ID, age and sex of the patient were collected. In addition, the Special Reimbursement Register was used to identify patients diagnosed with narcolepsy.

As the study was based only on administrative, secondary register data, under Finnish law no Ethics Board approval was required [37]. As the register holder, SII approved the use of the data for the current study.

### Cohort definition

**Fig. 1:**
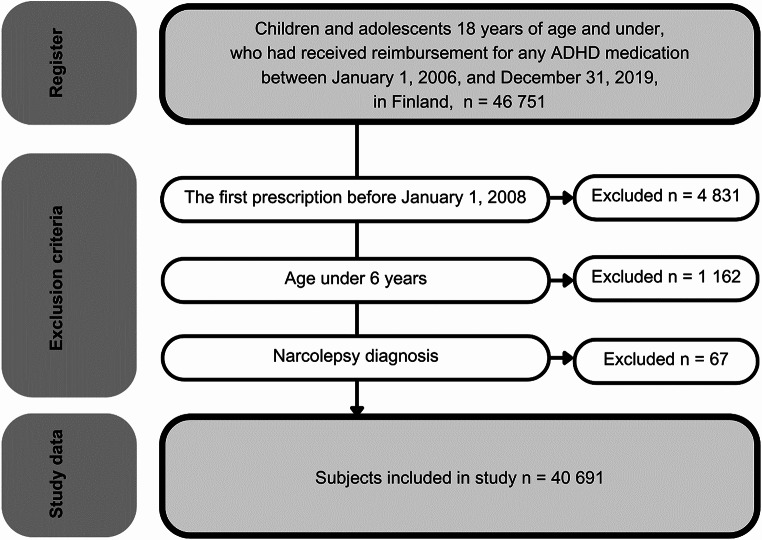
Cohort definition

The original register data included all children and adolescents 18 years of age and under, who had received reimbursement for ADHD medication purchase between January 1, 2006, and December 31, 2019, in Finland (*n* = 46751) (Fig. 1).

Treatment initiation was defined as the date of the first reimbursed purchase for any ADHD medication. To ascertain true naïve use a 2-year rolling wash-out period was implemented. In alignment with the Finnish clinical guideline for the treatment of ADHD [20], we excluded all children under the age of six (*n* = 1162), since these children are recommended to receive pharmacological treatment for ADHD only under very specific circumstances. Also, to prevent misclassification, we excluded all subjects with narcolepsy (*n* = 56), since narcolepsy is an alternative treatment indication for methylphenidate.

### Duration of treatment

We utilized repetitive purchases as a proxy for treatment continuation. Considering the episodic fashion of ADHD medication use [27], we allowed a cut-off up to 365 days between purchases.

The duration of treatment was determined in days, calculated as the period between the first and last purchase dates plus an additional 90 days. This approach rested on the assumption that the last redeemed purchase covered a period of a maximum of 90 days, aligning with the reimbursement policy of NHI, which only allows single purchases to cover a maximum of three months medication use.

### Statistical methods

Categorical variables were summarized with frequencies and percentages. Because of the skewed distribution of the age-variable in the data, medians with lower (Q1) and upper quartiles (Q3) were reported. The Kruskal-Wallis test was used to compare median ages at initiation between sexes. The study population was categorized into four age groups (6–8, 9–12, 13–15 and 16–18 years) based on the subject’s age at the time of the first reimbursed ADHD medication purchase.

Since the data included censored durations, the durations of ADHD medication treatment were estimated as Kaplan-Meier survival times. Medians along with both quartiles were reported with the corresponding 95% confidence intervals. The survival curves by sex and age group were compared using the log rank test. Cox proportional-hazards models were used to estimate associations between sex and age groups with discontinuation of ADHD medicine treatment. The hazard ratios were reported with 95% confidence intervals (95% CI).

To address the concern of possible changes in clinical practices over time and a large number of early-censored durations, the analysis was repeated for the subgroup of subjects who initiated ADHD medication treatment before January 1, 2016. Additionally, to understand the effect of our definition of treatment duration, a sensitivity analysis was conducted using both 90 and 180 days as cut-offs.

We set the level of statistical significance at 0.05. All analyses were conducted using SAS (SAS software for Windows version 9.4, SAS Institute Inc, Cary, NC). Kaplan-Meier graphs were created in R version 4.2.2 (R Core Team, 2022) utilizing survival (version 3.5-3) and survminer (version 0.4.9) packages.

## Results

In total, 40,691 children and adolescents aged 6–18 years purchased their first ADHD medication between January 1, 2008, and December 31, 2019. The median age at the initiation of ADHD medication was 9 years (Q1 = 8, Q3 = 12), with boys representing 77.4% of the initiators (*n* = 31502). The median age for boys was 9 years (Q1 = 7, Q3 = 12) and for girls 11 years (Q1 = 8, Q3 = 15) (*p* <.0001).

**Fig. 2:**
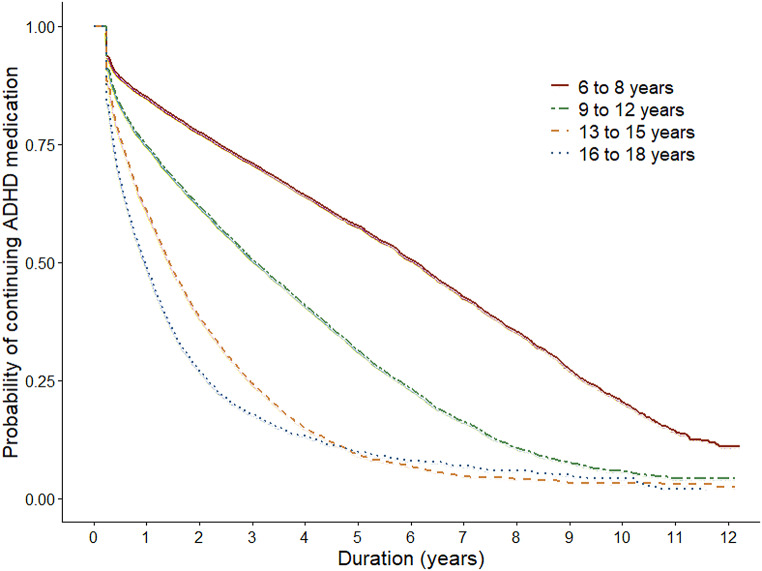
Kaplan-Meier curves illustrating the time to discontinuation of ADHD medication treatment, stratified by age group

**Fig. 3:**
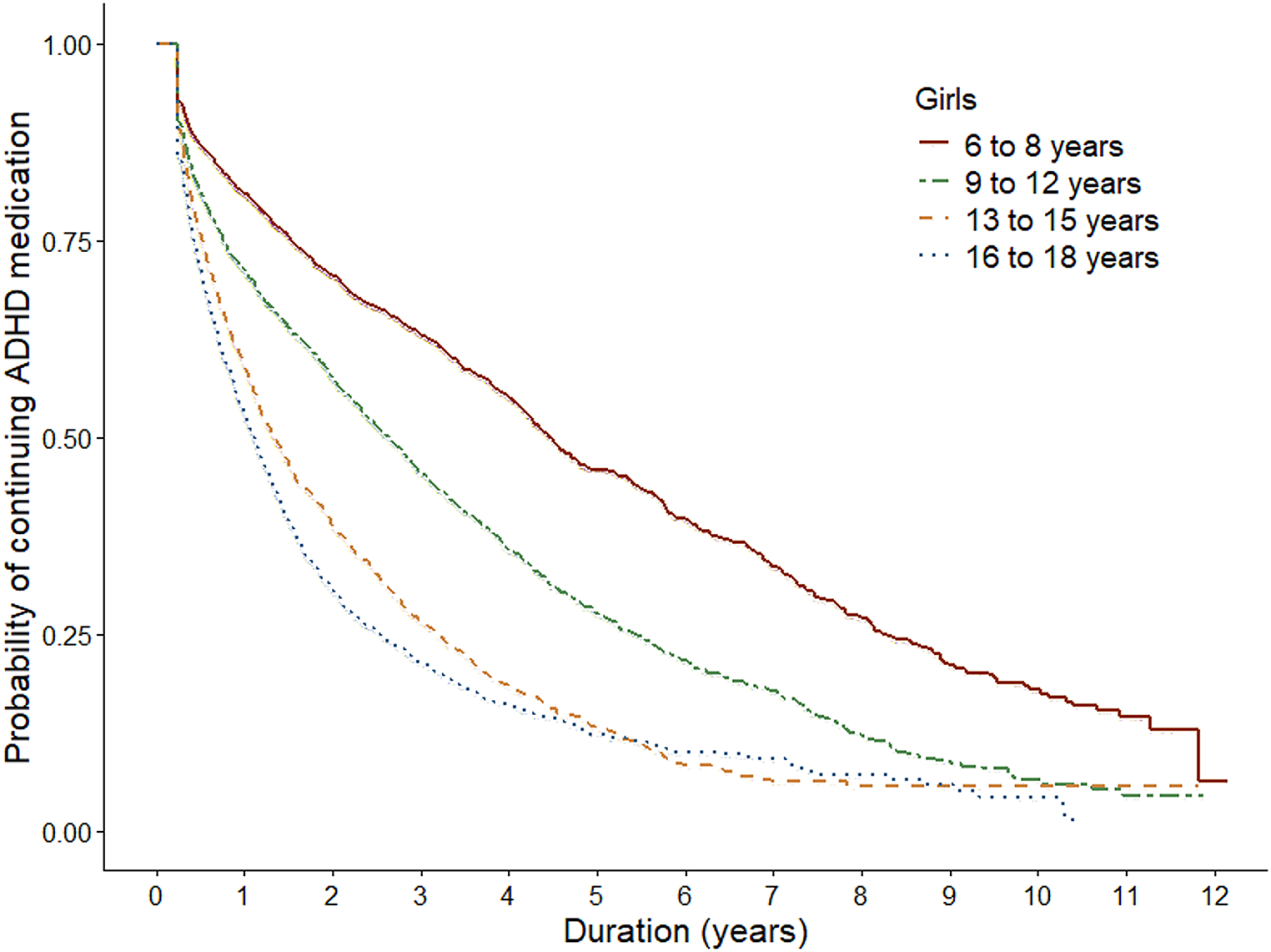
Kaplan-Meier curves illustrating the time to discontinuation of ADHD medication treatment among girls, stratified by age group

**Fig. 4:**
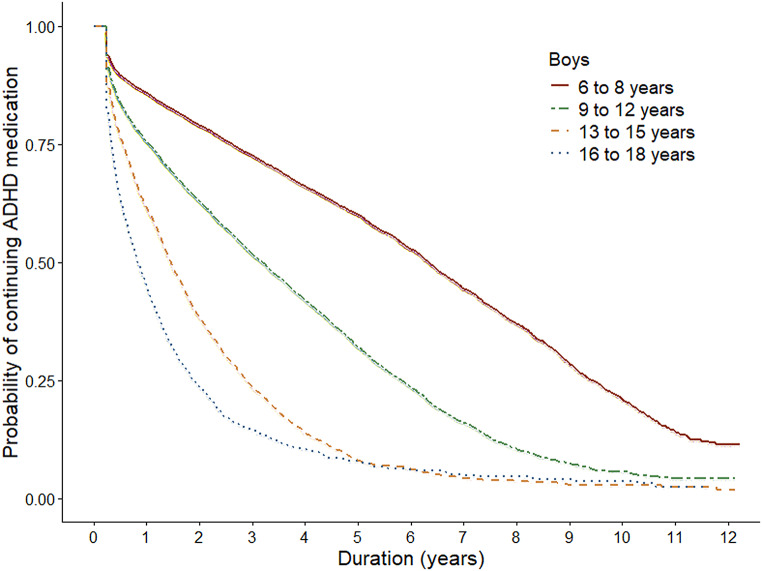
Kaplan-Meier curves illustrating the time to discontinuation of ADHD medication treatment among boys, stratified by age group

The median duration of the treatment with ADHD medication was 3.2 years (95% CI 3.2, 3.3) (Table 1; Fig. 2). One fourth of children and adolescents discontinued treatment within one year (Q1 = 1.0 year, 95% CI 0.9, 1.0). Conversely, one fourth continued treatment for at least 6.8 years (Q3 = 6.8 years, 95% CI 6.7, 7.0). Both sex and age were strongly associated with the duration of treatment (*p* <.0001, *p* <.0001 and *p* <.0001): boys tended to present with longer treatment duration than girls and the younger the subject, the longer the duration of use (Table 1; Figs. 3 and 4). The longest duration of treatment was observed among boys aged six to eight years (median = 6.3 years, 95% CI 6.2, 6.5, Q1 = 2.6 years, 95% CI 2.5, 2.7, Q3 = 9.4 years, 95% CI 9.2, 9.6).

The proportion of uncensored subjects who had only a single purchase as their initial ADHD medication treatment was 9.1% (95% CI 8.8, 9.4, *n* = 3093/33931).

**Table 1:** Median, Q1 and Q3 quartile durations (years) of ADHD medication treatment among Finnish children and adolescents aged 6–18 years. Hazard ratios with the statistical significance pertain to the likelihood of discontinuation of ADHD medication treatment compared to the reference group

	*n*	%_Group_ [95% CI]	Median [95% CI]	Q1 [95% CI]	Q3 [95% CI]	HR [95% CI]	*p*
Overall	40 691		3.2 [3.2, 3.3]	1.0 [0.9, 1.0]	6.8 [6.7, 7.0]		
Sex							< 0.0001
Girls	9 189	22.6 [22.1, 23.0]	2.2 [2.2, 2.4]	0.7 [0.7, 0.7]	5.6 [5.4, 5.8]	1.3 [1.3, 1.4]	
Boys	31 502	77.4 [77.0, 77.8]	3.5 [3.5, 3.6]	1.1 [1.1, 1.1]	7.1 [7.0, 7.2]	ref	
Girls							< 0.0001
6–8	2744	29.9 [28.9, 30.8]	4.5 [4.3, 4.7]	1.5 [1.4, 1.7]	8.3 [7.8, 8.9]	ref	
9–12	2789	30.4 [29.4, 31.3]	2.6 [2.5, 2.8]	0.8 [0.7, 0.8]	5.4 [5.1,0.5.8]	1.6 [1.5, 1.7]	
13–15	1752	19.1 [18.3, 19.9]	1.3 [1.2, 1.5]	0.5 [0.5, 0.6]	3.2 [2.9, 3.5]	2.5 [2.3, 2.7]	
16–18	1904	20.7 [19.9, 21.6]	1.1 [1.0, 1.2]	0.4 [0.4, 0.5]	2.5 [2.2, 2.8]	2.9 [2.7, 3.1]	
Boys							< 0.0001
6–8	13 194	41.9 [41.3, 42.4]	6.3 [6.2, 6.5]	2.6 [2.5, 2.7]	9.4 [9.2, 9.6]	ref	
9–12	11 970	38.0 [37.5, 38.5]	3.2 [3.1, 3.3]	1.1 [1.0, 1.1]	5.8 [5.7, 6.0]	2.0 [2.0, 2.1]	
13–15	4463	14.2 [13.8, 14.6]	1.5 [1.4, 1.5]	0.6 [0.5, 0.6]	2.9 [2.8, 3.0]	3.9 [3.7, 4.0]	
16–18	1875	5.9 [5.7, 6.2]	0.8 [0.8, 0.9]	0.3 [0.3, 0.4]	1.9 [1.8, 2.1]	4.9 [4.9, 5.5]	

Discontinuation of ADHD medicine treatment was more common in the older age groups. The treatment was discontinued by the 9–12-year-olds 2.0 times more likely than in 6–8-year-olds (HR 2.0, 95% CI 1.9, 2.1), 3.6 times more likely in 13–15-year-olds (HR 3.6, 95% CI 3.5, 3.8) and 3.9 times more likely in 16–18-year-olds (HR 3.9, 95% CI 3.7, 4.2) (Table 1).

The median follow-up time was 3.8 years (min = 0, Q1 = 1.7, Q3 = 7.1, max = 12.0). At the end of follow-up, 44.6% of the subjects were still continuing treatment (*n* = 18144/40691). Among those with censored duration, the median follow-up time was 1.9 years (min = 0, Q1 = 0.8, Q3 = 4.1, max = 12.0). To evaluate the effect of early-censored durations, a sensitivity analysis was performed by selecting a subset of data from subjects who initiated ADHD medication treatment before January 1, 2016. For this the subgroup, the median follow-up time was 7.2 years (min = 4.0, Q1 = 5.5, Q3 = 7.2, max = 12.0, *n* = 19614). The recalculated durations were consistent with those obtained from the entire dataset, validating the use of the full dataset. Results from this sensitivity analysis are detailed in the supplementary material.

Additionally, the chosen cut-off of 365 days between purchases did not influence our findings. The median, Q1 and Q3 quartile durations were equal even with cut-off of 90 and 180 days as evidenced by a median time lapse of only 35 days between individual purchases (Q1 = 28, Q3 = 57). Furthermore, 88.4% of the purchases occurred within 90 days from the previous purchase and 99.5% the purchases occurred within 267 days.

## Discussion

In this population-based register study among Finnish children and adolescents we found that the average duration of medical ADHD treatment was over three consecutive years. In addition, every fourth subject continued ADHD medication treatment for at least seven years. The duration of ADHD medicine use was longer among those initiating pharmacological treatment in childhood when compared with those initiating as adolescents. Among boys and girls who initiated treatment at ages 6–8, the median duration of ADHD medication use was 6.3 and 4.5 years, respectively. In this group, one fourth continued treatment for at least 8 to 9 years.

Our results on ADHD treatment duration are in line with recent population-based studies that demonstrate that around 50% of those who initiate treatment in childhood are still continuing up to 5 years of follow-up [29, 32]. Based on our results, however, a substantial proportion of children continue treatment even longer. This could indicate that especially during the first years of our study period, the ADHD medication treatments in Finland were specifically targeted for children with more severe symptoms and thus more likely to continue treatment for several years. This notion is supported by the recent multinational study that found between country variation in treatment persistence with the highest persistence rates being observed in Denmark [32] where the prevalence of ADHD medication among children and adolescents has been similarly low as in Finland, when compared to other Nordic countries. In Finland, the administrative prevalence rates of ADHD medication treatment in 2018 were around 4–5% among boys and as low as 1% among girls [38]. However, in our recent study we showed the incidence of ADHD treatment in Finland increasing, in particular, among 6–8-year-old boys and at such a pace that raises concern on potential targeting of medical treatments currently also to children with subthreshold symptoms [39]. Our findings also corroborate the existing empirical evidence showing that the age at onset of therapy plays an important role. Adolescents are clearly more likely to discontinue treatment earlier than children [32]. Possible explanations in “real-world” clinical settings may arise from medication-related factors such as differences in effectiveness and tolerance [9]. Furthermore, the younger the child the greater the parents’ influence on treatment continuation.

Additionally, we found the duration of ADHD medicine treatment to be longer among boys when compared to girls. Although the difference was somewhat modest, this finding is conflicting with prior studies that have not showed clear differences between the sexes [29, 32]. For reasons unclear, Finnish boys have been significantly more often treated with ADHD medication than girls [23, 38, 39]. In our data, 77.4% of the incident users were boys. Interestingly, there is some evidence that boys display better improvements in ADHD symptoms when using methylphenidate compared with girls, which may reflect sex differences in pharmacokinetics [40]. However, this issue goes beyond the scope of our study and is a topic for further research.

Considering the above, our study is the first to report on extended duration of ADHD medication use among 6–8-year-olds going well beyond the data available from randomized controlled trials [19, 21]. For the regulatory approval of ADHD medications, the European Medicines agency requires pharmaceutical companies to establish clinical safety with a study covering at least 1 year of follow-up [41]. Longer prospective, post-marketing safety studies are recommended as part of the risk management plan. Accordingly, the recently published results from the ADDUCE study covered safety data for methylphenidate over a 2-year period in relation to growth and development, psychiatric and neurological health and cardiovascular function in children and adolescents [19]. The study found no serious, life-threatening adverse events during the follow-up and the observed reduction in growth was not statistically significant. However, due to the uncontrolled, naturalistic nature of the study, there was no randomization of subjects into comparison groups limiting conclusions on causality. By contrast, the Multimodal Treatment of Attention Deficit Hyperactivity Disorder study (MTA) showed stimulants to diminish growth in the range of 1 to 2 cm from predicted adult height and especially among children who were on higher and consistently administered doses of stimulants [42]. The effects diluted by the third year of treatment, but without evidence of rebound growth. The findings prevailed in the subsequent follow-up analysis indicating that 16 years of consistent stimulant treatment of children with ADHD in the MTA was associated to shorter adult height and an increase in weight and BMI [25]. Still, the evidence base for unexpected and rare adverse events with ADHD medications is lacking.

Capturing a rare medicinal adverse event with a frequency of 1: 1 000–10 000 in a randomized controlled clinical study would require a study population of thousands to tens of thousands and is, therefore, not feasible nor ethical in pediatric ADHD. However, large administrative registries can be investigated years after marketing authorisation with computerized statistical approaches and valid pharmaco-epidemiological methods. Based on our results on the amounts of children being exposed to ADHD medications throughout their whole elementary school phase, it would seem appropriate to conduct large, high quality post authorization safety studies of sufficiently long follow-up to capture potential, rare and unexpected adverse events occurring with ADHD medications in this vulnerable patient group of children and adolescents.

Our study is the first to capture long term use of pediatric ADHD medications, going beyond 5 years of follow-up. Furthermore, our study utilizes nationwide population-based registry data only available in the Nordic countries. Still our study has several limitations. First, we used reimbursed purchases as a proxy for consumption [35]. Second, our results on the duration of treatment might be underestimated: While we aimed to control for censored data and misclassification bias with sufficient follow-up available for all subjects, a substantial proportion of subjects still continued with treatment at the end of follow-up. Furthermore, we may have overestimated discontinuation considering that re-use may have occurred also after the 365 days cut-off period. Third, we were not able to discern the clinical features of subjects initiating their ADHD treatment nor on their non-pharmacological co-treatments or relevant clinical outcomes. Fourth, we captured only data from the pre-pandemic era. Recent data show that the prevalence of ADHD medication use among children in Finland has continued to increase since 2020 [43].

There are also other, local factors potentially influencing continuation of ADHD medication treatment that may limit the generalizability of our findings to other European countries. Since the adoption of the updated National Core Curriculum for Basic Education in 2016, schools have undergone a fundamental reform in Finland, including new emphasis on promoting digital competencies. Schools have, however, had varying digital resources and many schools have also promoted the use of a child’s own smart device in digital teaching [44], with variability in local practices to hinder or restrict inappropriate use during class [45, 46]. Excessive exposure to screens is a known risk factor for inattentive or hyperactive symptoms [47–52]. Furthermore, the specialist shortage of child psychiatrists and child neurologists with neurodevelopmental disabilities training [53] is likely to affect the quality of care available for children and adolescents with ADHD symptoms [17]. Since 2017, the Finnish Current Clinical Care guideline on ADHD has, however, recommended the diagnostic and treatment decision making to primarily take place in the primary health care sector and specialists should be consulted only if needed [20, 54]. In fact, showed in our previous study, the incidence of ADHD medicine treatment among children and adolescents has increased exponentially in Finland during our study years [39]. Thus, instead of undertreatment, the local shortage of professionals with in-depth training on childhood neurodevelopmental disabilities could, instead, hinder appropriate discontinuation and cessation of ADHD medication treatment in the long run. Since the core ADHD symptom severity tends to decline as children mature, many clinical guidelines on ADHD recommend to assess annually whether the patient still requires medication treatment [17, 20]. After the first 1‒2 years of medication the risk for relapse also seems to decrease [55]. This indicates that many patients successfully treated could discontinue treatment and still maintain the benefits [17].

In conclusion, the duration of ADHD medication treatment among children in the real-world clinical setting goes well beyond the data available from randomized controlled trials and extends for several years especially among young boys. This warrants for high quality follow-up of treatment in clinical practice including repetitive weighing of the expected benefits and potential risks of medication for every treated child and at the population level.

## Electronic supplementary material

Below is the link to the electronic supplementary material.

Supplementary Material 1

## Data Availability

Due to data protection regulations of the secondary use of administrative, individual-level register data in Finland, the authors do not have the permission to make the data supporting the current findings openly available. Interested parties may however apply for permission to access the data from the Finnish Social and Health Data Permit Authority (Findata) or Insurance Institution of Finland (SII).
