# Supplementary material for: Duration of ADHD medication treatment among Finnish children and adolescents ‒ a nationwide register study
Source: Eur Child Adolesc Psychiatry. 2025 May 7;34(10):3151–60. doi: 10.1007/s00787-025-02735-4 (PMC12592317; doi:10.1007/s00787-025-02735-4)
Supplement: Supplementary file 1 — Supplementary Material 1 [file 787_2025_2735_MOESM1_ESM.docx]

Supplementary table 1. Median, Q1 and Q3 quartile durations (years) of ADHD medication treatment among Finnish children and adolescents aged 6-18 years. Sensitivity analysis data includes subjects who initiated ADHD medication treatment between January 1, 2008, and December 31, 2015. The final analysis data includes subjects who initiated ADHD medication treatment between January 1, 2008, and December 31, 2019.

|  | Data | n | Median  [95% CI] | Q1  [95% CI] | Q3  [95% CI] |
| --- | --- | --- | --- | --- | --- |
| Overall | Sensitivity | 19614 | 3.6 [3.4, 3.6] | 1.0 [1.0, 1.0] | 7.0 [6.8, 7.2] |
|  | Final | 40 691 | 3.2 [3.2, 3.3] | 1.0 [0.9, 1.0] | 6.8 [6.7, 7.0] |
| Sex |  |  |  |  |  |
| Girls | Sensitivity | 3820 | 2.6 [2.4, 2.8] | 0.6 [0.6, 0.8] | 6.0 [5.8, 6.2] |
|  | Final | 9 189 | 2.2 [2.2, 2.4] | 0.7 [0.7, 0.7] | 5.6 [5.4, 5.8] |
| Boys | Sensitivity | 15794 | 3.8 [3.6, 3.8] | 1.2 [1.0, 1.2] | 7.2 [7.0, 7.2] |
|  | Final | 31 502 | 3.5 [3.5, 3.6] | 1.1 [1.1, 1.1] | 7.1 [7.0, 7.2] |
| Girls |  |  |  |  |  |
| 6–8 | Sensitivity | 1255 | 4.8 [4.4, 5.4] | 1.6 [1.4, 2] | 8.4 [8.0, 9.0] |
|  | Final | 2744 | 4.5 [4.3, 4.7] | 1.5 [1.4, 1.7] | 8.3 [7.8, 8.9] |
| 9–12 | Sensitivity | 1252 | 2.8 [2.6, 3.0] | 0.8 [0.6, 1] | 5.6 [5.2, 6.0] |
|  | Final | 2789 | 2.6 [2.5, 2.8] | 0.8 [0.7, 0.8] | 5.4 [5.1,.5.8] |
| 13–15 | Sensitivity | 672 | 1.2 [1.0, 1.4] | 0.4 [0.4, 0.6] | 3.4 [3.0, 3.6] |
|  | Final | 1752 | 1.3 [1.2, 1.5] | 0.5 [0.5, 0.6] | 3.2 [2.9, 3.5] |
| 16–18 | Sensitivity | 641 | 1.0 [1.0, 1.2] | 0.4 [0.4, 0.4] | 3.0 [2.6, 3.6] |
|  | Final | 1904 | 1.1 [1.0, 1.2] | 0.4 [0.4, 0.5] | 2.5 [2.2, 2.8] |
| Boys |  |  |  |  |  |
| 6–8 | Sensitivity | 6434 | 6.4 [6.4, 6.6] | 2.8 [2.6, 3.2] | 9.4 [9.2, 9.6] |
|  | Final | 13194 | 6.3 [6.2, 6.5] | 2.6 [2.5, 2.7] | 9.4 [9.2, 9.6] |
| 9–12 | Sensitivity | 6137 | 3.4 [3.2, 3.6] | 1.2 [1.0, 1.2] | 6.0 [5.8, 6.2] |
|  | Final | 11970 | 3.2 [3.1, 3.3] | 1.1 [1.0, 1.1] | 5.8 [5.7, 6.0] |
| 13–15 | Sensitivity | 2344 | 1.4 [1.4, 1.6] | 0.6 [0.4, 0.6] | 3.0 [3.0, 3.2] |
|  | Final | 4463 | 1.5 [1.4, 1.5] | 0.6 [0.5, 0.6] | 2.9 [2.8, 3.0] |
| 16–18 | Sensitivity | 879 | 0.8 [0.8, 1.0] | 0.4 [0.4, 0.4] | 2.2 [2.0, 2.4] |
|  | Final | 1875 | 0.8 [0.8, 0.9] | 0.3 [0.3, 0.4] | 1.9 [1.8, 2.1] |
